# Complete Genome Sequences of Five Rabies Virus Strains Obtained from Domestic Carnivores in Liberia

**DOI:** 10.1128/mra.01047-21

**Published:** 2022-01-20

**Authors:** Stephanie Mauti, Garmie Voupawoe, Simon Bonas, Varney Kamara, Hervé Bourhy, Morgane Gourlaouen, Paola De Benedictis, Jakob Zinsstag, Laurent Dacheux

**Affiliations:** a Institut Pasteur, Université de Paris, Lyssavirus Epidemiology and Neuropathology Unit, National Reference Center for Rabies and WHO Collaborating Center for Reference and Research on Rabies, Paris, France; b Swiss Tropical and Public Health Institute, Basel, Switzerland; c University of Basel, Basel, Switzerland; d Leon Quist Ledlum Central Veterinary Laboratory, Ministry of Agriculture, Monrovia, Republic of Liberia; e FAO Reference Centre for Rabies, Istituto Zooprofilattico Sperimentale delle Venezie, Legnaro, Padua, Italy; KU Leuven

## Abstract

As in other African countries, canine rabies is endemic in Liberia. However, data concerning the genetic diversity of rabies virus isolates circulating in this country remain limited. We report here the complete genome sequences of five rabies viruses obtained from domestic animals. All of them belonged to subgroup H within the Africa 2 clade.

## ANNOUNCEMENT

Rabies virus (RABV) is the main etiological agent of rabies, an acute and always fatal form of encephalomyelitis which can potentially affect all mammalian species. This zoonotic virus belongs to the prototype species Rabies lyssavirus within the genus *Lyssavirus*, family *Rhabdoviridae* (order *Mononegavirales*) (1). Rabies viruses circulating in dogs are the main cause of human rabies, with an estimated 59,000 deaths worldwide each year, especially in Asia and Africa (2). As in other sub-Saharan countries, canine rabies remains endemic in Liberia (3). However, available data about the genetic diversity of RABV isolates circulating in this country remain limited.

**FIG 1:**
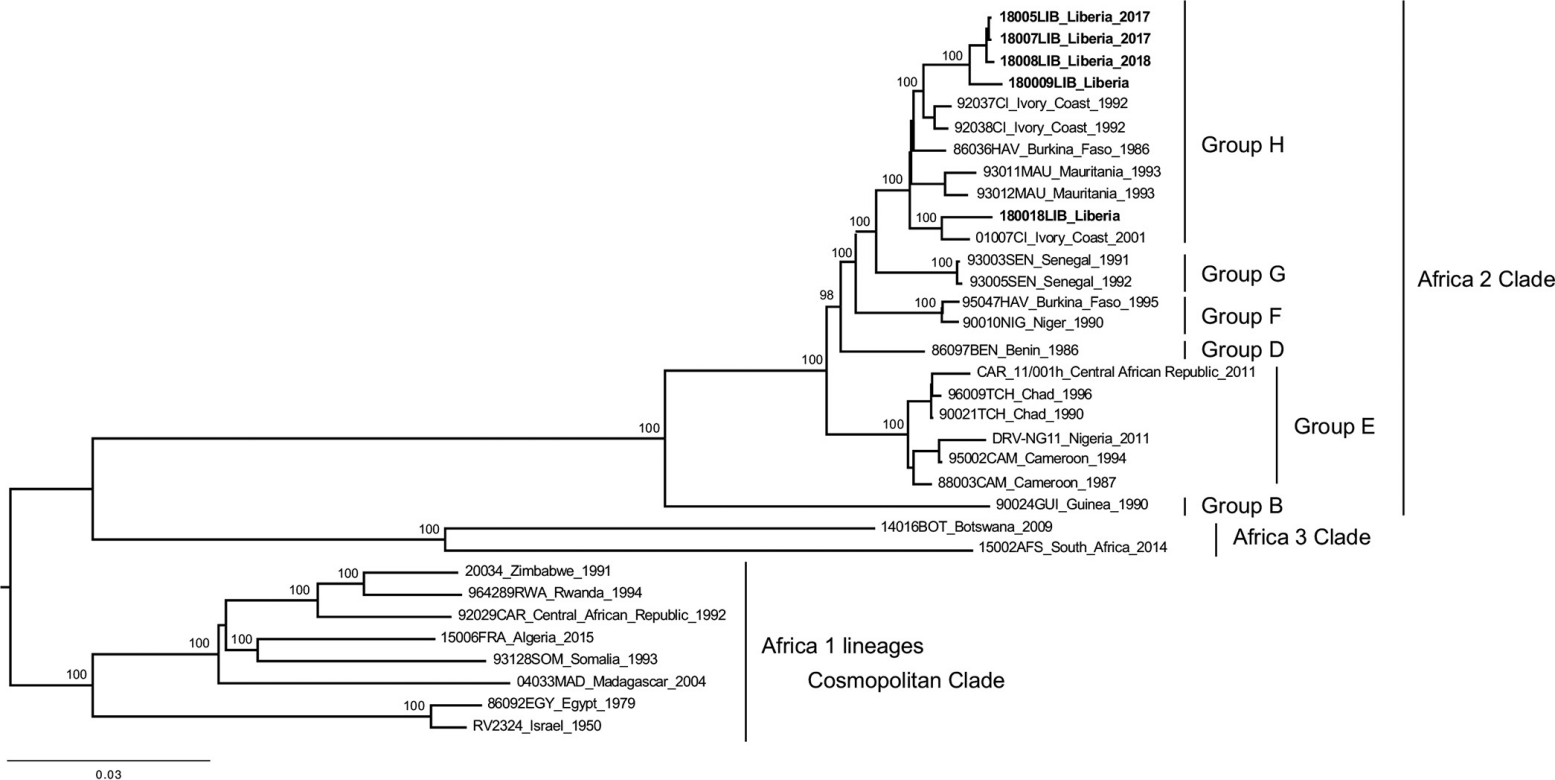
Phylogenetic analysis of the five RABV strains from Liberia and different representative African strains. The tree was based on the nearly complete genome sequences (11,800 to 11,804 nucleotides [nt]) and constructed using the maximum‐likelihood approach, based on the generalized time‐reversible model proportion of invariable sites plus the gamma‐distributed rate heterogeneity (GTR+I+Γ4), utilizing subtree pruning and regrafting (SPR) branch‐swapping, as estimated in PhyML version 3.0 (13) with Smart Model Selection (http://www.atgc-montpellier.fr/phyml-sms/). The robustness of individual nodes was estimated using 100 bootstrap replicates. The different phylogenetic clades, lineages, and groups have been previously described (3, 15, 16). Groups A and C were missing from the Africa 2 clade, due to the lack of complete genome sequences available. Only bootstrap values of ≥90 are indicated. The scale bar indicates the number of nucleotide substitutions per site.

Brain samples collected from four dogs and one cat suspected of rabies were collected from different regions of Liberia in 2017 and 2018, within the framework of a joint effort program to strengthen rabies surveillance in the country (Table 1) (3). All the samples were confirmed positive for rabies by fluorescence antibody test (FAT) (4) and by a modified version of a rapid immunochromatographic diagnostic test (RIDT) (5). For four samples, RNA was extracted locally from brain biopsy specimens (approximatively 0.5 cm^3^ each) using the Direct-zol RNA miniprep kit (Zymo Research) and then purified using Agencourt RNAClean XP beads (Beckman Coulter) at a 1:1.8 ratio. The last sample was extracted at Institut Pasteur using TRIzol reagent (Invitrogen) from an FTA card (Whatman FTA card technology; Sigma-Aldrich) impregnated with ground brain material as previously described (Table 1) (6). The five RNA samples were processed for next-generation sequencing (NGS) as previously described (7–9). Briefly, an rRNA depletion step was first carried out using Terminator 5′‐phosphate‐dependent exonuclease (Epicentre Biotechnologies). After purification, depleted RNA was reverse-transcribed into cDNA using Superscript III reverse transcriptase (Invitrogen), and double-stranded DNA (dsDNA) was synthesized as already described (7–9). Finally, dsDNA libraries were constructed using the Nextera XT DNA library preparation kit (Illumina) and sequenced using a 2 × 150-nucleotide (nt) paired-end strategy on the NextSeq 500 platform (7–9). NGS data were analyzed using *de novo* assembly and mapping (both using CLC Assembly Cell; Qiagen), with a dedicated workflow built on the Institut Pasteur Galaxy platform (7–10). Contig sequences were assembled to produce the final consensus genome using Sequencher version 5.2.4 (Gene Codes Corporation). The quality and the accuracy of the final genome sequences were checked after a final mapping step of the original cleaned reads and visualized using Tablet (11). The leader and trailer sequences were verified after alignment with genetically close and available complete genomes (Fig. 1; Table 1). Identification of the open reading frames was performed using SnapGene software version 5.3.2. The nucleotide identity was determined using Ident and Sim software implemented in the Sequence Manipulation Suite (https://www.bioinformatics.org/sms2/ident_sim.html) (12). Maximum likelihood (ML) phylogenetic analysis was performed on the nearly complete genome sequences (11,800 to 11,804 nt) of the five RABV strains and different representative African strains using PhyML (13), after multiple alignment performed using ClustalW version 2.1 (14) implemented in the Institut Pasteur Galaxy platform (10). The ML phylogenetic tree was visualized using FigTree (http://tree.bio.ed.ac.uk/) (Fig. 1). All protocols were performed according to the manufacturer’s instructions, and all tools were run with default parameters, unless otherwise specified.

**TABLE 1:** Description of the genome sequences of the five rabies virus strains obtained from Liberian domestic carnivores

Virus	Host	Animal status	Location	Yr of collection	Support^*a*^	Total no. of reads	No. of mapped reads (%)	Avg coverage (×)	Genome nucleotide length (bp)	GC content (%)	ORF nucleotide length (aa)^*b*^					GenBank accession no.	SRA accession no.
											N	P	M	G	L		
18005LIB	Cat	Owned	Margibi	2017	Beads	4,317,876	934,436 (21.6)	11,567.36	11,922	45	1,353 (450)	891^*c*^ (296)	609 (202)	1,575 (524)	6,384 (2,127)	OK135144	SRX12176932
18007LIB	Dog	Owned	Montserrado	2017	Beads	2,228,096	4,593 (0.2)	56.95	11,923	45	1,353 (450)	891^*c*^ (296)	609 (202)	1,575 (524)	6,384 (2,127)	OK135145	SRX12176933
18008LIB^*d*^	Dog	Owned	Montserrado	2018	Beads	5,132,556	12,554 (0.2)	155.23	11,885^*e*^	45	1,353 (450)	891^*c*^ (296)	609 (202)	1,575 (524)	6,384 (2,127)	OK135146	SRX12176934
18009LIB	Dog	Owned	Lofa	NA^*f*^	FTA	1,916,466	25,596 (1.3)	316.18	11,923	45	1,353 (450)	894 (297)	609 (202)	1,575 (524)	6,384 (2,127)	OK135147	SRX12176935
18018LIB	Dog	NA	NA	NA	Beads	7,209,068	826,598 (11.5)	10,135.10	11,923	45	1,353 (450)	894 (297)	609 (202)	1,575 (524)	6,384 (2,127)	OK135148	SRX12176936

a Total RNA was extracted in Liberia from strains 18005LIB, 18007LIB, 18008LIB, and 180185LIB (recovered from brain biopsy specimens [approximatively 0.5 cm^3^] from separate animals) and purified using Agencourt RNAClean XP beads (Beckman Coulter) at a 1:1.8 ratio following the manufacturer’s instructions, with the exception of the last resuspension step in nuclease-free water. The dried beads with RNA were shipped at a cold temperature with icepacks to Institut Pasteur (Paris), where they were resuspended in 30 μL nuclease-free water. Strain 18009LIB was sent to Institut Pasteur using an FTA card (Whatman FTA card technology; Sigma-Aldrich) impregnated with ground brain material and then extracted using TRIzol reagent (Invitrogen).

b ORF, open reading frame; aa, amino acid.

c P ORF with premature stop codon (missing the last amino acid C).

d Strain 18008LIB was also partially sequenced at Istituto Zooprofilattico Sperimentale delle Venezie (IZSVe-FAO Reference Center for Rabies) in Italy, and the sequence was found to be identical to the one obtained by Institut Pasteur Paris (IPP).

e Genome with incomplete 5′ UTR (untranslated region) (leader sequence missing 38 nucleotides).

f NA, not available.

The genome sequences presented the five canonical genes encoding the nucleoprotein (N), phosphoprotein (P), matrix protein (M), glycoprotein (G), and RNA polymerase (L) (Table 1). The leader and trailer sequences were 58 and 70 nucleotides long, respectively. The transcription initiation (TI) signal AACA and the transcription termination (TTP) TGA_7_ was observed for all the genes, except for the G gene, which presented the AGA_7_ motif for TTP. Three sequences presented a premature stop codon in the P gene. The nucleotide identity between four of the genome sequences was high (>99.1%), whereas strain 18018LIB was slightly more divergent (>97.5%). Genetic analysis confirmed that they clustered together in group H within the Africa 2 clade (3, 15, 16) (Fig. 1).

### Data availability.

The complete genome sequences of the five rabies viruses from Liberia were deposited at GenBank under the accession numbers OK135144, OK135145, OK135146, OK135147, and OK135148 and the BioProject accession number PRJNA763029.
